# Trends in Medical Aid in Dying in Oregon and Washington

**DOI:** 10.1001/jamanetworkopen.2019.8648

**Published:** 2019-08-09

**Authors:** Luai Al Rabadi, Michael LeBlanc, Taylor Bucy, Lee M. Ellis, Dawn L. Hershman, Frank L. Meyskens, Lynne Taylor, Charles D. Blanke

**Affiliations:** 1 Oregon Health & Science University, Portland; 2 SWOG Statistics and Data Management Center, Seattle, Washington; 3 MD Anderson Cancer Center, Houston, Texas; 4 Columbia University, New York, New York; 5 University of California, Irvine, Chao Family Comprehensive Cancer Center, Orange; 6 University of Washington, Seattle; 7 SWOG Group Chair’s Office, Portland, Oregon

## Abstract

**Importance:**

The combined 28 years of data of medical aid in dying (MAID) between Oregon (OR) and Washington (WA) are the most comprehensive in North America. No reports to date have compared MAID use in different US states.

**Objective:**

To evaluate and compare patterns of MAID use between the states with the longest-running US death with dignity programs.

**Design, Setting, and Participants:**

A retrospective observational cohort study of OR and WA patients with terminal illness who received prescriptions as part of their states’ legislation allowing MAID. All published annual reports, from 1998 to 2017 in OR and from 2009 to 2017 in WA, were reviewed. A total of 3368 prescriptions were included.

**Main Outcomes and Measures:**

Number of deaths from self-administration of lethal medication vs number of prescriptions written.

**Results:**

A combined 3368 prescriptions were written in OR and WA, with 2558 patient deaths from lethal ingestion (76.0%). Of the 2558 patients, most were male (1311 [51.3%]), older than 65 years (1851 [72.4%]), and non-Hispanic white (2426 [94.8%]). The most common underlying illnesses were cancer (1955 [76.4%]), neurologic illness (261 [10.2%]), lung disease (144 [5.6%]), and heart disease (117 [4.6%]). Loss of autonomy (2235 [87.4%]), impaired quality of life (2203 [86.1%]), and loss of dignity (1755 [68.6%]) were the most common reasons for pursuing MAID. Time between drug intake to coma ranged from 1 to 660 minutes and time from drug intake to death ranged from 1 to 6240 minutes. In the 1557 patients for whom rates of complications were reported, 1494 (96.0%) did not experience a complication (592 of 626 [94.6%] in OR and 902 of 931 [96.8%] in WA). Eight patients (<0.5%) regained consciousness after drug ingestion in OR. Annual rates per year for percentage of patients who received a prescription ingesting the prescribed medication ranged from 48% to 87%, with no significant time trend in OR (adjusted odds ratio per year, 1.01; 95% CI, 0.99-1.02; *P* = .59) but with an increase over time in WA (adjusted odds ratio per year, 1.13; 95% CI, 1.08-1.19; *P* < .001). In both OR and WA there were increases in the number of patient deaths due to MAID per 1000 deaths over time.

**Conclusions and Relevance:**

In this study, MAID results in Oregon and Washington were similar, although MAID use measured as a percentage of patients prescribed lethal medications and then self-administering them increased only in WA. Most patients who acquired lethal prescriptions had cancer or terminal illnesses that are difficult to palliate and lead to loss of autonomy, dignity, and quality of life.

## Key Points

**Question:**

Are patterns of medical aid in dying (MAID) use similar in Oregon and Washington?

**Findings:**

In this cohort study of 3368 prescriptions for MAID in Oregon and Washington, most patients in both states were insured, non-Hispanic white individuals with some level of college education, with cancer being the most common underlying illness prompting MAID request. Annual percentage of patients per year who were prescribed medication and ingested it did not change in Oregon but increased over time in Washington, and in both states there were increasing deaths due to MAID per 1000 deaths over time.

**Meaning:**

With the exception of use of prescribed medication, MAID patterns are similar in Oregon and Washington.

## Teaser

This cohort study evaluates and compares patterns of the use of medical aid in dying in Oregon and Washington State.

## Introduction

The Oregon Death With Dignity Act, passed in 1994, legally permits terminally ill adults (age ≥18 years) with legal Oregon (OR) residency to make the voluntary informed choice to obtain a physician’s prescription for oral drugs to end life.^1^ In 2008, Washington state (WA) approved similar legislation, making it the second US state to legalize medical aid in dying (MAID).^2^ Today, MAID has become more widely adopted and is officially permitted in 8 jurisdictions in the United States (OR [1994], WA [2008], Montana [2009], Vermont [2013], California [2015], Colorado [2016], District of Columbia [2017], and Hawaii [2019]) and will be adopted in New Jersey on August 1, 2019.

Legislative processes inherent within the passage of the Death With Dignity Acts in both OR and WA ensure safeguards for patients seeking MAID, including guaranteeing procedural guidelines for oversight by local and state agencies.^3^ Such precautionary measures include requiring the patient to place 3 separate oral and written requests with a built-in waiting period and a required assessment to assess decisional capacity in any case in which it is suspect.^1,2^ However, differences in the framework of MAID legislation between each state may result in different outcomes, and, to our knowledge, data on the implementation and uniformity of this practice have never been compared between 2 US states. Notably, policy researchers in Canada identified significant interprovincial differences in Canadian MAID program processes and practice.^4^

Since enactment of MAID legislation in OR and WA, both states mandate publication of an annual statistical report with information pertaining to patients and physicians who participate in MAID. Here, we review the combined 28 years of data on MAID from 2 states with the longest history of established MAID legislation to determine major patterns of use, compare demographic trends over time, and evaluate whether they are consistent across both jurisdictions.

## Methods

### Data Collection

All annual reports published by the Oregon Health Authority and the Washington State Department of Health, from 1998 to 2017 in Oregon and from 2009 to 2017 in Washington, were reviewed in their entirety by 4 of us (L.A.R., M.L., T.B., and C.D.B.). The data sets generated and analyzed during the current study are available on the Oregon Health Authority^5^ and Washington State Department of Health^6^ websites. This study qualified for institutional review board exemption by the Oregon Health and Science University Office of Integrity as the study used publicly available data in such a manner that participants could not be identified. This study followed the Strengthening the Reporting of Observational Studies in Epidemiology (STROBE) reporting guideline for cohort studies.^7^

### Statistical Analysis

Characteristics of those dying as a result of orally ingested lethal medication(s) were first collected independently by state, and then combined if they differed by less than 5%. Time trends for deaths from ingesting lethal medication vs number of prescriptions were analyzed using logistic regression.^8^ The time trends for assessing the changes in deaths per 1000 population were analyzed with Poisson regression where population figures were obtained from state health departments.^5,6^ Final death numbers were not available for WA for 2015 and 2016, so they were estimated based on mean increases over the last 3 years from WA.

The age distributions of individuals who used MAID were calculated for all years in WA and from 2006 onward for OR given differing age group definitions in earlier OR reporting. A χ^2^ test statistic was used to test differences in age distributions for the 2 programs. Trends in changes in the age distribution between programs were assessed based on logistic regression where patients were divided in 2 age groups, 75 years and older vs younger than 75 years. All *P* values reported are 2-sided, with a level of significance of *P* < .05.

## Results

Over the combined 28 years of publicly available data in OR and WA, 3368 prescriptions were written, with 2558 patients (76.0%) dying after they ingested medication. Persons using MAID were approximately equally distributed by sex (1311 [51.3%] male), non-Hispanic white individuals were most commonly represented (2426 patients [94.8%]), most patients were older than 65 years (1851 [72.4%]), and the majority had received some college education (1830 [71.5%]) and were insured (2264 [88.5%]) at the time of request (Table 1). Age distribution was not statistically significant between states (Table 2).

**Table 1.** Patient Characteristics for Oregon and Washington^a^

Baseline Characteristic	Participants, No. (%) (N = 2558)
Sex	
Male	1311 (51.2)
Female	1247 (48.8)
Age range, y	20-102
Race	
White	2426 (94.8)
Other	101 (3.9)
Unknown	31 (1.2)
Some college education	1830 (71.5)
Medically insured	2264 (88.5)
Oregon	1157 (90.8)
Washington	1107 (81.2)
Underlying illness	
Cancer	1955 (76.4)
Neurological condition, eg, amyotrophic lateral sclerosis	261 (10.2)
Lung disease, eg, chronic obstructive pulmonary disease	144 (5.6)
Cardiac disease, eg, congestive heart failure	117 (4.6)
Other	117 (3.0)
Enrolled in hospice	1943 (76.0)

^a^ Data include cases from 1998 to 2017 in Oregon and from 2009 to 2017 in Washington.

**Table 2.** Age Distribution Among Medical Aid in Dying Participants in Oregon and Washington

Age, y	Participants, %	
	Oregon	Washington
18-44	2.5	2.5
45-54	4.5	6.0
55-64	19.7	20.0
65-74	30.8	30.4
75-84	25.6	23.0
≥85	16.9	18.0

Most patients who received prescriptions had a diagnosis of cancer (1955 [76.4%]), while the remaining had neurologic illness (261 [10.2%]), lung disease (144 [5.6%]), heart disease (117 [4.6%]), or other illnesses (77 [3.0%]). The most frequent reasons for requesting MAID were loss of autonomy (2235 [87.4%]), decreasing ability to participate in pleasurable activities or impaired quality of life (2203 [86.1%]), and loss of dignity (1755 [68.6%]). Four percent of patients received referrals for psychiatric examinations. Enrollment rates in hospice were high, with 87.8% and 64.2% of patients enrolled at the time of death in OR and WA, respectively.

A total of 2075 patients (81%) died at home, and prescribers were present in 247 cases (9.7% total; 187 [14.7%] in OR and 60 [4.7%] in WA). Time between drug intake and coma ranged from 1 to 660 minutes and time from drug intake to death ranged from 1 to 6240 to minutes. From data reported in the OR data set, median time from ingestion to coma was 5 minutes and time from ingestion to death was 25 minutes. Notably, in 638 drug recipients (50.0%) in OR, it was unknown whether a complication occurred. This is due to a 2010 amendment on the follow-up questionnaire that only allowed information regarding time of and circumstances surrounding death to be reported if a clinician was present at time of death. Among 1557 patients for whom complication rates were reported, 1494 (96%) did not experience a complication (592 of 626 [94.6%] in OR and 902 of 931 [96.8%] in WA). Eight patients (<0.5%) awoke in OR after drug ingestion.

### Time Trend Analyses

We evaluated key measures of the 2 programs. Annual rates per year for percentage of patients ingesting medication ranged from 48% to 87%, with no significant time trend in OR (adjusted odds ratio per year, 1.01; 95% CI, 0.99-1.02; *P* = .59) but with an increase over time in WA (adjusted odds ratio per year, 1.13; 95% CI, 1.08-1.19; *P* < .001).

We used the participant age distribution partitioned at 75 years or older vs younger than 75 years to evaluate changes over time. There was no significant change in the fraction of individuals over time in OR (adjusted odds ratio, 1.02; 95% CI, 0.98-1.05; *P* = .40). In WA there was a nonsignificant increase (adjusted odds ratio, 1.05; 95% CI, 1.00-1.10; *P* = .05).

The trends for programs were also calibrated against total state population deaths. In both OR and WA there were increases in the numbers of patients using MAID per 1000 deaths over time. Estimates and regression lines from fitted Poisson regression models, using logarithm link to outcome, are seen in the Figure. The *P* values for trend for WA and OR were both less than .001 with a greater rate of increase in WA with a significant interaction test statistic (OR, 0.94; 95% CI, 0.91-0.96; *P* < .001). While annual estimates give some suggestion that the rate of increase is slowing for both programs in the last 2 years, it will take several more years of follow-up data for this to be confirmed.

**Figure.**
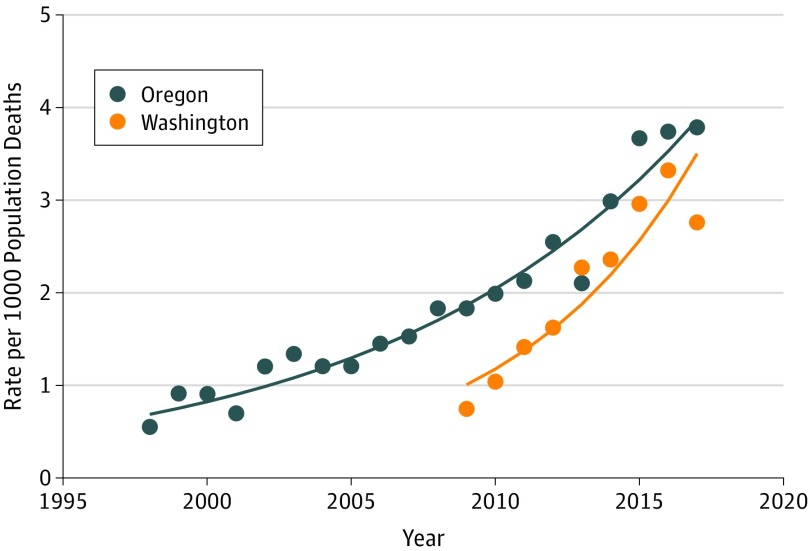
Rate of Medical Aid in Dying per 1000 State Mortality

## Discussion

Previous reports have detailed MAID use in OR, the state with the longest-running death with dignity program.^9^ States such as WA have modeled their MAID legislation after OR’s, but outcomes have not been extensively detailed or compared. Canada has legalized MAID across the entire country, but each province or territory independently sets up its policies and procedures, and results to date have demonstrated wide variability.^4^ However, the outcomes of MAID legislation in OR and WA have been remarkably similar. The population of terminally ill individuals pursuing MAID is approximately equally distributed across sex and predominantly consists of persons aged 65 years and older and individuals diagnosed with cancer (Table 1 and Table 2). The vast majority are non-Hispanic white individuals (94.8%) with some level of college education (71.5%), and 88.5% were insured by public or private programs. These data reinforce the belief that MAID has not been directed toward traditionally vulnerable populations based on age, race/ethnicity, level of educational attainment, or insurance status. In addition, the Figure illustrates that with the increasing age of legislation, there has been a corresponding increase in MAID use in both OR and WA. We can hypothesize that this is a result of similar MAID legislation in both states and supported by increased public awareness of such legislation.

The most notable differences between both states include the number of patients enrolled in hospice at the time of death, with a greater number of patients enrolled at the time of death in OR (87.8%) vs WA (64.2%). This may be partially explained by the slightly higher number of insured patients at the time of enrollment (90.8% in OR vs 81.2% in WA).

Our data set supports the overall safety and reliability of the lethal medications used in MAID. Among patients who ingested the lethal drugs and were evaluable for complications, only 4% experienced complications, the most common being difficulty ingesting or regurgitation of the lethal drug. Intervention by emergency medical services after the lethal drug was ingested, information that is only collected by WA, did not occur over a 10-year period. Short median times to unconsciousness and death prevent undue and lengthy suffering and reflect the efficacy of drugs used in MAID.

Reasons patients choose to pursue MAID include loss of autonomy, impaired quality of life, inadequate pain control, and, in a small percentage, financial concerns. The reasons underlying MAID may be representative of the larger population of individuals facing the end of life and should be formally studied. Additionally, MAID research to date has been mostly descriptive.^9,10^ Formal interventional trials are being proposed, including a study assessing the effect of mandatory referral for treatment of depression on MAID follow-through (Robert S. Krouse, MD, email communication, May 8, 2019). There is significant room for further integration of palliative care, social support services, and case management in end-of-life decision-making with the intent of increasing the options available to those facing a terminal disease. In the 2019 OR legislative session, 3 measures were introduced pertaining to MAID, including amendments that would redefine the term *ingestion* to include any method by which a patient introduces a medication into his or her body, eliminate the 6-month period that currently defines terminal disease (Oregon House Bill 2903), define the term *self-administer* (Oregon House Bill 2217), and create an exception to the defined waiting periods for patients who have extremely limited life expectancies (Oregon Senate Bill 579).

### Limitations

The quality of our data is limited by the fact that the underlying reasons for requesting MAID were not collected from patients but supplied by physicians in the form a follow-up questionnaire. As such, there is no way of ascertaining whether the enquiries were comprehensive.

In 2010, an amendment to the follow-up questionnaire in OR only allowed information regarding time of and circumstances surrounding death to be reported if a clinician was present at time of death. This has resulted in approximately half of patients having data collected mentioning complications, which consequently runs a risk of underreporting complications and makes it difficult to completely assess the safety surrounding MAID. Some opponents of MAID estimate higher complication rates and advocate for greater physician involvement, specifically the use of physician-assisted euthanasia.^11^

## Conclusions

In this study, MAID results in Oregon and Washington were similar, although MAID use measured as a percentage of patients prescribed lethal medications and then self-administering them increased only in WA. Most patients who acquired lethal prescriptions had cancer or terminal illnesses that are difficult to palliate and lead to loss of autonomy, dignity, and quality of life. Concerns that MAID would unintentionally target socially disadvantaged patients have not materialized, as evidenced by the data presented in this article. States considering MAID legalization may see similar results if they model their rules on those put into place in the US Pacific Northwest.
